# Multi-Functional Applications of Hydrogel Delivery Systems in Inflammatory Bowel Disease: Drug Delivery, Anti-Inflammation, and Intestinal Repair

**DOI:** 10.3390/polym17111430

**Published:** 2025-05-22

**Authors:** Yuhui Sun, Juefei Wu, Jiaqi Zan, Zekun Li, Luyun Liu, Gang Ding

**Affiliations:** School of Stomatology, Shandong Second Medical University, Weifang 261053, China; sunyh03xy@163.com (Y.S.); hawlily2697439@163.com (J.W.); z1369089383@163.com (J.Z.); zekun_lee@163.com (Z.L.); liuluyun0120@163.com (L.L.)

**Keywords:** inflammatory bowel disease, ulcerative colitis, Crohn’s disease, hydrogel, delivery system

## Abstract

Inflammatory bowel disease (IBD) represents a chronic inflammatory disorder of the gastrointestinal tract with a multifactorial etiology that remains incompletely elucidated. Accumulating evidence implicates dysregulation of the intestinal micro-ecosystem, aberrant neuroimmune interactions, and compromised epithelial barrier integrity as key contributors to IBD pathogenesis. While oral administration remains the predominant therapeutic approach, the acidic gastric milieu and enzymatic catabolism markedly compromise drug efficacy. Consequently, selecting an optimal drug delivery method has become a pressing issue in IBD management. As a drug delivery platform, hydrogels, distinguished by their favorable biocompatibility, biodegradability, and injectability, can shield drugs from the harsh gastrointestinal environment. This review offers an innovative and comprehensive analysis of the interactions among various hydrogel application forms, delivery routes, and loaded substances, summarizing the advantages of different types of hydrogels in terms of their anti-inflammatory properties and maintenance of intestinal flora homeostasis, as well as discussing the limitations of current hydrogel deliver systems and looking to the future.

## 1. Introduction

Inflammatory bowel disease (IBD), a group of gastrointestinal disorders, is primarily characterized by chronic inflammation occurring in various regions of the gastrointestinal tract. It encompasses two major subtypes: ulcerative colitis (UC), which primarily occurs between the rectum and the proximal colon and affects the mucosal and submucosal layers; and Crohn’s disease, which can affect any part of the digestive tract, from the mouth to the anus, and is progressive and destructive in nature. The clinical symptoms of IBD include bleeding, weight loss, abdominal pain, diarrhea, bloody stools, etc., severely impairing the quality of life [1]. The pathogenesis of IBD is complex and involves multiple factors, including microbiota, immunity, genetics, and environmental factors. In a healthy intestinal environment, the intestinal mechanical barrier, microbial balance, and immune homeostasis jointly maintain intestinal stability [2]. In intestines affected by IBD, macrophage polarization leads to the secretion of pro-inflammatory cytokines and the production of large amounts of reactive oxygen species (ROS), leading to intestinal mucosa damage and pathogen invasion, breaking the immune balance and destroying the intestinal epithelial barrier [3]. Furthermore, microbial imbalance may play an important role in triggering IBD, which further aggravates the immune response and destroys the intestinal barrier [4].

The main drug delivery methods for treating IBD consist of oral administration, rectal administration, and intravenous injection [5]. However, oral medications are susceptible to the effects of gastrointestinal pH and enzymes, which may reduce the drug’s activity and bioavailability, and long-term and high-dose application is prone to systemic side effects [6]. Rectal administration can enhance the local effect of drugs, but it has a short retention time in the body and affects the therapeutic effect [7]. Although injectable administration can circumvent the first-pass effect and enterohepatic circulation issues, the significant pain and operational complexity reduce patient compliance. Therefore, the development of an appropriate drug delivery system to achieve precise delivery and optimal therapeutic outcomes has become especially urgent.

Hydrogel is a polymer with a three-dimensional network structure. It is widely used in the medical field due to its hydrophilicity, reusability, and excellent biocompatibility, forming a drug delivery system with a slow-release function to extend the duration of action of drugs [8].

This review not only covers single drug delivery but also delves into the mechanisms of different bio-based and responsive hydrogels delivering mesenchymal stem cells (MSCs), growth factors, probiotics, and other substances. Ultimately, we scrutinize the challenges and future directions of hydrogels in IBD clinical therapy, and propose clinically-oriented solutions, which will offer theoretical underpinnings and facilitate the translational application of hydrogels in IBD clinical therapy.

## 2. Application Forms of Hydrogel Delivery Systems in IBD

With the continuous optimization of hydrogel-based drug delivery methods, their application forms have also been updated. Injectable hydrogel, micro hydrogel, and composite hydrogel delivery systems are common applications [9].

### 2.1. Injectable Hydrogel

The rutin-loaded guanosine phenylborate hydrogel, composed of guanosine (G), benzene-1,4-diboronic acid (BDBA), and rutin (R), is a pH/ROS dual-responsive hydrogel (GBR). The boronate ester bonds result in a storage modulus (G′) lower than the loss modulus (G″) at room temperature under shear, indicating increased viscosity, gelation, and excellent self-healing ability [10]. The GBR hydrogel exhibited a rapid and nearly complete drug release profile under a weak acidic condition and ROS overexpression microenvironment, in contrast to its more restrained drug release in a simulated physiological environment, thereby enabling targeted drug delivery (Table 1). The results of the studies indicated that rectal administration of the GBR hydrogel facilitated colonic mucosal repair and reduced the extent of inflammation in the treatment of IBD.

The excessive stimulation of inflammation can trigger the coagulation cascade and lead to the formation of microthrombi, while tissue hypoxia and ischemia further complicate the damage. Ag^+^ is crosslinked with heparin (HEP) and functionalized bovine serum albumin (BSA) through electrostatic interaction to form a HEP-Ag-BSA hydrogel which is injectable, self-healing, antithrombotic and anti-inflammatory. When hydrogels of different colors formed by HEP-Ag-BSA are placed side by side, their boundaries disappear within 5 min, indicating remarkable self-healing properties. In vitro clotting tests reveal that both the HEP-Ag-BSA group and the HEP group exhibit better coagulation effects. In vivo experiments also confirm that heparin exerts thrombolytic effects, reducing the tissue damage and systemic circulation impact caused by thrombi, thus facilitating tissue repair. Immunohistochemical analysis indicated that rectal administration of HEP-Ag-BSA markedly reduced myeloperoxidase activity and interleukin-6 (IL-6) expression, while simultaneously enhancing syndecan-1 and basic fibroblast growth factor (bFGF) immunoreactivity, in comparison to the dextran sulfate sodium (DSS)-induced IBD models [11].

### 2.2. Microhydrogels

Hydrogel microspheres and microcapsules each have their unique advantages and applications. Hydrogel microspheres, with high biocompatibility and multifunctionality, show great potential in tissue engineering and drug delivery [15]. Additionally, microcapsules, with their core-shell structure and rapid response characteristics, hold significant value in precise drug delivery and targeted therapy [16].

As the center of glutathione peroxidases, selenium (Se) deficiency can exacerbate mucosal damage and inflammation in UC [17]. Mannose-functionalized Se nanoparticles (M-SeNPs) can enhance the regulatory effect on intestinal epithelial cells and alleviate inflammation by reprogramming macrophages [18]. Embedding M-SeNPs into colon-targeted hydrogels forms chitosan(CS)/delivery system containing alginate (SA)-MSe hydrogel microbeads, which can inhibit the nuclear factor kappa-light-chain-enhancer of activated B cells (NF-κB) signaling pathway, counteracting the inflammation-induced reduction of glutathione peroxidase expression in colon-related cell lines like NCM460, and thus alleviating DSS-induced colitis [12]. Furthermore, the specific combination of mannose and the surface of intestinal epithelial cells enables C/S-MSe hydrogel beads to have precise targeting and realize drug delivery (Table 1).

### 2.3. Hydrogel Compound Drug Delivery System

Due to their unique physical and chemical properties and versatility, composite hydrogels have attracted much attention in biomedicine, environmental engineering, energy engineering, and other fields, and exhibit additional functional properties by incorporating NPs, polymers, biomolecules, and other materials [19,20]. Both metal-organic frameworks (MOFs) and nanoneedles have the ability to clear ROS. Inulin hydrogel is adhesive, and can prolong the drug retention time and help maintain the intestinal flora balance [21]. Using the hydrothermal method, inulin hydrogels encapsulating CuCl_2_ and olsalazine were combined with MOF and nanoneedles to form Cu_2_(Olsa)/Gel, which has good stability and drug release (Table 1). In vitro studies have shown that Cu_2_(Olsa)/Gel reduces the level of ROS in macrophages and reduces the migration of H_2_O_2_ to intestinal epithelial cell-6 cells. In addition, after the application of Cu_2_(Olsa)/Gel in vivo, the ratio of *Firmicutes/Bacteroidetes* also approached normal levels, promoting the restoration of the intestinal microbiota balance [13]. Pectin-CS (PC) and polyacrylamide (PAM) hydrogels (PC/PAM hydrogels) have dual pH and enzyme responses and can be used for colon targeting and controlled release drug loading of budesonide (Bud) (Table 1). According to the results of the gel fraction and swelling ratio of the hydrogel, the proportion of PC/PAM was optimized to achieve the two-phase release of drugs [14].

## 3. Delivery Methods of Hydrogels in IBD

A multi-functional platform based on the hydrogel delivery system can protect the drug from oral, rectal, and injection administration, so that the drug can target the inflammatory site, enhance the concentration of the drug in the inflammatory site, and improve the bioavailability. Moreover, the physical and chemical properties of the hydrogel, such as its swelling behavior, stability, and responsiveness to different environments, can prolong the duration of drug action at the site of inflammation, thereby enhancing the drug’s antioxidative and anti-inflammatory effects [22] (Table 2).

### 3.1. Oral Route

#### 3.1.1. Oral Administration

Currently, oral administration is the main method for the treatment of IBD with drugs such as 5-aminosalicylic acid (5-ASA) [32], nanoenzymes [33], and rhein (Rh) [34], among other free drugs that can be delivered directly via the oral route, but they are easily affected by the gastrointestinal environment.

Compared to delivering free curcumin (CUR) or emodin (EMO) alone, researchers have innovatively developed a pH-responsive dual-drug delivery system (sodium alginate (SA)) of CUR/EMO nanohydrogels (NE@SA) [23]. By leveraging the synergistic effects of CUR and EMO to inhibit the NF-κB pathway, CUR/EMO NE@SA not only significantly reduces the levels of pro-inflammatory cytokines like tumor necrosis factor-α (TNF-α) but also reverses epithelial barrier damage, achieving colon-targeted drug delivery and sustained drug release (Table 2).

IBD not only affects the gut but also increases the risk of extra-intestinal manifestations such as anxiety and depression. Dysbiosis can impair the nervous system through the microbiota-gut-brain axis, triggering neuroinflammation and leading to neuronal damage [34]. A Rh hydrogel based on Spirulina platensis (SP@Rh-gel) not only can improve the stability of EMO and inhibit the production of pro-inflammatory cytokines, but can also reduce the formation of M1 microglia in the hippocampus, effectively alleviating depression and anxiety behavior in experimental mice [24].

Intestinal microorganisms are essential for maintaining intestinal homeostasis, but the activity of probiotics, such as *Escherichia coli Nissle 1917* (EcN), will be impaired in the acidic environment after oral administration [35,36]. EcN was loaded into a supramolecular self-assembled novel nitroreductase (NTR) unstable peptide hydrogel (EcN@Gel), in order to improve the tolerance of probiotics to acidic environments and enzymes such as gastric acid and bile acid (Table 2). Compared with the control group, the hydrogel can improve the survival rate of EcN, control the release of probiotics, reduce the content of pro-inflammatory cytokines, and promote the repair of the intestinal barrier [25]. In another study, an inulin hydrogel loaded with a polypyrrole (PPy) nanoenzyme and antifibrosis pirfenidone (PFD) was used. Oral administration can prolong the action time of the nanoenzyme, maintain the balance of intestinal flora, and promote the healing of colitis [26].

The recent development of CAT@ALG-PDA microreactors, which were created by encapsulating catalase (CAT) within a calcium alginate hydrogel, modifying it with a polydopamine (PDA) shell, forming a powder, and further encapsulating the powder in an enteric capsule, provides a promising therapeutic strategy for clinical application, which preserves its integrity and enzymatic activity for 24 h in artificial colonic fluid and commences degradation in artificial gastric fluid within 2 h. Moreover, the therapeutic efficacy of CAT@ALG-PDA surpasses that of adalimumab, which was usually used in the treatment of IBD patients in clinics, resulting in lower Disease Activity Index scores, greater preservation of colon length, and more significant reduction in pro-inflammatory cytokine expression levels [37].

#### 3.1.2. Intragastric Administration

Compared to oral administration, intragastric administration circumvents the impact of oral cavity enzymes on drugs, allowing them to rapidly enter the gastrointestinal tract and exert their effects swiftly. However, this method also exposes drugs to the acidic environment. Natural plant polysaccharides, such as Brasenia schreberi (BS), are coated with a special polysaccharide hydrogel (BS mucilage). After intragastric administration of a BS hydrogel in a DSS mouse model, the antioxidant effect of the BS hydrogel was achieved by up-regulating the expression of catalase and reducing the expression of oxidative gene markers. In addition, the BS hydrogel can also inhibit the abundance of bacteria associated with colorectal cancer, such as *Acutalibacter* and *Christensenella* [38]. In another study, a gelated peritoneal macrophage (GPM) that EcN can specifically recognize was formed through intracellular hydrogel technology, and intragastric administration could improve the utilization and adhesion of EcN. In vivo studies showed that intragastric administration of GPM-EcN significantly enhanced the utilization and adhesion of EcN, and inhibited intestinal fibrosis and inflammatory macrophage infiltration, playing an important role in its anti-inflammatory effect and promoting intestinal barrier repair [39].

### 3.2. Rectal Administration

After rectal administration, the retention time of the drug in the intestine is prolonged by using the adhesion of hydrogels, so as to improve the curative effect [40]. Hyaluronic acid (HA) can target macrophages, while CS-based drug-loaded thermosensitive hydrogels enable in situ drug delivery to the colon [41]. A novel delivery system combining Puerarin-loaded NPs with thermosensitive hydrogels (Pur@HA-SH-zein NPs in hydrogel) can adhere better to the colon after rectal injection. The immunofluorescence results showed that C6@HA-SH-zein NPs exhibit the strongest green fluorescence under the action of HA and can bind with F4/80 red fluorescence, indicating that HA has selectivity towards macrophages [27].

A notable characteristic of UC is that it primarily affects the distal colon [42]. Administration of hydrogel enemas can avoid the first-pass effect and ensure drug concentration in the distal colon. Quercetin (Que)-loaded guanosine borate supramolecular hydrogel (named GBQ hydrogel) has good injectability when strain increases to 600% (G′ < G″). The results show that the GBQ hydrogel is more effective than Me and Que alone, as dynamic boronic ester bonds, sensitive to ROS/pH, promote Que release in the inflammation, repairing the mucosal barrier and exerting stronger anti-inflammatory and antioxidant effects [28] (Table 2). Additionally, dexamethasone (DEX), bFGF, and L-alanyl-L-glutamine (ALG) loaded into a hydrogel form a mechanical self-healing hydrogel. After rectal administration, it can significantly inhibit the toll-like receptor 4 (TLR4)-NF-κB signaling axis and reduce the M1 macrophage-mediated immune response, and at the same time, it has strong mechanical properties and tissue adhesion, so that the drug can act on the ulcer site for a long time, promoting mucosal integrity and functional recovery [29].

Anal administration may cause infection and patient discomfort, but drugs can act directly on the anus. Therefore, hydrogels are required to have good mechanical and adhesive properties, as well as biocompatibility and support with tissues. Using arginine-glycine-aspartic acid hydrogels to encapsulate human amniotic epithelial stem cells for anal administration preserved the stem cell characteristics of stem cells while promoting the secretion of specific microRNA-23a-3p to reduce the expression of TNF receptor1. In vitro studies have shown that miR-23a-3p inhibits the phosphorylated NF-κB signaling pathway in colon epithelial cells by directly targeting TNF receptor1. This novel method avoids the risks associated with immunosuppressive treatments by restoring intestinal epithelial homeostasis, demonstrating the potential of hydrogel-based anal drug delivery in the treatment of IBD [43].

### 3.3. Injection Administration

#### 3.3.1. Subcutaneous Injection

Subcutaneous injection is a novel method of hydrogel administration compared with oral and rectal administration. 1,4-dihydrophenathrolin4-one-3-carboxylic acid (DPCA), one kind of small molecule prolyl hydroxylase (PHD) inhibitor, can up-regulate hypoxia-inducible factor one-α and has a regenerative effect. However, under normoxic conditions, it can be rapidly degraded by PHD enzyme, inhibiting the activity of PHD [44]. Therefore, a PEG-DPCA hydrogel was subcutaneously injected into the posterior part of the neck of a DSS-induced colitis mouse model. In the short term, both preventative DPCA or restorative DPCA could promote epithelial recovery and showed reduced inflammation [45]. In another study, adipose tissue-derived human MSCs were pretreated and loaded into injectable in situ-crosslinking alginate (Alg) hydrogels, and combined with HEP-coated beads loaded with IFNγ to prepare a licensing hydrogel, which not only reduced the frequency of administration, but also extended the biological activity of the MSCs, significantly alleviating colonic inflammation [46].

#### 3.3.2. Parenteral Injection

Parenteral injection of hydrogels can more accurately inject into the lesion site, while reducing the drug in the blood circulation. Compared with implantation of human placenta-derived MSCs (hP-MSCs) alone, co-transplantation of a co-grafting CS-based injectable hydrogel with solidified IGF-1C structural domain polypeptide (CS-IGF-1C) and hP-MSCs for parenteral injection can improve the survival rate of hp-MSCs and show better efficacy in the treatment of IBD [47].

## 4. Various Substances Delivered by Hydrogel

### 4.1. Medication

The treatment of IBD primarily involves pharmacological therapy, with common anti-inflammatory drugs including 5-ASA [48], CUR [49], and proanthocyanidins [50], which possess anti-inflammatory, antioxidant, and immunomodulatory effects, helping to reduce the release of inflammatory cytokines, and promote the repair of the intestinal mucosa.

Me is mainly used in mild-to-moderate cases of UC with severe adverse effects. Recent studies have identified a hydrogel delivery system of MC/HA-Me that can be formed by combining hyaluronic acid-mesalazine (HA-Me) with methylcellulose (MC), showing that gelation occurs in a simulated physiological environment at MC concentrations of 5–7 wt% and that the cumulative release of the drug is approximately 50% within 3 h, with a sustained release within 12 h. In addition, HA-Me was able to bind specifically to the CD44 receptor at the site of colonic inflammation, achieving colonic inflammation targeting, and significantly inhibiting the production of inflammatory cytokines [51].

### 4.2. Mesenchymal Stem Cells (MSCs)

**Figure 1:**
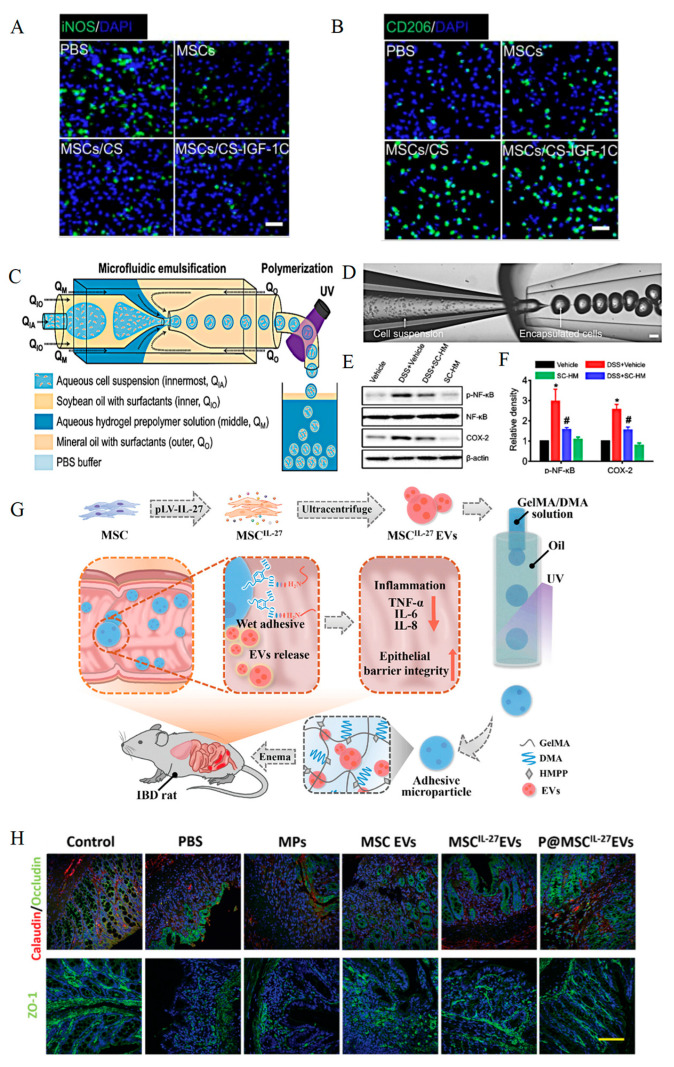
The formation process of a hydrogel delivery system loaded with stem cells, and its abilities regarding anti-inflammation and repair of the intestinal epithelial barrier. (**A**,**B**) Immunofluorescence showed a significant increase in M2 polarization of macrophages after co-transplantation of hP-MSCs and CS-IGF-1C. Reprinted from Theranostics, vol.10, Cao, Xiaocang et al. [47], Copyright (2020). (**C**,**D**) Schematic diagram of SC-HM formation. Reprinted from Journal of controlled release: official journal of the Controlled Release Society, vol. 347, Kim, Do-Wan et al. [16], Copyright (2022). (**E**,**F**) Changes in phosphorylation of the NF-κB signaling pathway and expression levels of COX-2 after oral administration of SC-HM. * *p* < 0.01 vs. Vehicle, ^#^ *p* < 0.05 vs DSS + Vehicle. Reprinted from Journal of controlled release: official journal of the Controlled Release Society, vol. 347, Kim, Do-Wan et al. [16], Copyright (2022). (**G**) The formation process, administration mode, mechanism of action, and therapeutic effect of P@MSC^IL−27^EVs. Reprinted from Advanced science (Weinheim, Baden-Wurttemberg, Germany), vol. 10, Nie, Min et al. [56], Copyright (2023). (**H**) After treatment with P@MSC^IL−27^EVs, the expressions of Occludin/Calaudin and ZO-1 increase, and the intestinal epithelium gradually repairs. Reprinted from Advanced science (Weinheim, Baden-Wurttemberg, Germany), vol. 10, Nie, Min et al. [56], Copyright (2023).

MSCs, which are easily isolated and obtained, are a type of pluripotent stem cell with multipotent differentiation capabilities and low immunogenicity, making them ideal materials in the fields of tissue engineering and regenerative medicine [52]. MSCs can inhibit the development of inflammation through a series of signaling pathways, such as NF-κB signaling pathways [53,54]. As a scaffold material, hydrogels can load and protect stem cells, guide them to the lesion site, and make them grow in a favorable microenvironment, thus enhancing the potential of MSCs in treating IBD [55]. Previous studies have shown that hydrogels can protect the activity of stem cells by binding to the C domain peptide of insulin-like growth factor-1. Co-transplantation of CS-IGF-1C and hP-MSCs can promote the secretion of PGE_2_ by MSCs, and enhance the M2 polarization of macrophages and the secretion of IL-10 [47] (Figure 1A,B).

As indicated by existing studies, hydrogel microcapsules with a thin oil layer are capable of encapsulating bioactive materials within their individual core regions, such as hydrogel microcapsules that encapsulate umbilical cord-derived mesenchymal stem cells into thin oil layers (SC-HMs) (Figure 1C,D). When observed at 24 h and 48 h, the viability of UCB-MSCs encapsulated in SC-HM was 94.3 ± 2.65% and 90.27 ± 6.24%, respectively. Western blot analysis revealed that SC-HM can markedly suppress the phosphorylation of the NF-κB pathway and down-regulate Cyclooxygenase-2 (COX-2) expression (Figure 1E,F). Additionally, SC-HM can restore the ratio of *Firmicutes/Bacteroidetes* to nearly normal levels, thus effectively maintaining the gut microbiota balance [16]. In another study, the MSC-derived extracellular vesicles with a high expression of IL-27 (MSC^IL−27^EVs) were encapsulated in dopamine methacrylamide-modified hydrogels, combined with GelMA to form a composite hydrogel (D-GM hydrogel). Microcarriers (G-DM MPs) created by microfluidic technology were utilized due to their good wet adhesiveness, with an adhesion rate of nearly 18% (Figure 1G). Immunofluorescence staining showed that G-DM MPs loaded with MSC-EVs (P@MSC^IL−27^EVs) could increase ZO-1 and occludin expression levels, resulting in a more intact intestinal epithelial structure [56] (Figure 1H).

### 4.3. Nanoenzymes

The excessive accumulation of reactive oxygen and nitrogen species at the site of colonic inflammation can lead to epithelial barrier disruption and dysbiosis of the gut microbiota [57]. PPy nanozymes can clear ROS and regulate oxidative stress, while PFD can inhibit intestinal fibrosis. Encapsulating PPy nanoenzymes and PFD with an inulin hydrogel (PPy/PFD@Inulin gel) not only protects the activity of nanoenzymes, demonstrating excellent H_2_O_2_ clearance and cell protection abilities, but also achieves the dual therapeutic effects of treating IBD and inhibiting intestinal fibrosis [26].

Cerium oxide (CeO_2_) NPs with Ce^3+^ and Ce^4+^ sites exhibit superoxide dismutase and catalase activity, which can effectively and stably clear ROS and reduce damage to inflammatory sites [58]. CCNZ&Alg is formed by a gel of CeO_2_ nanoenzymes coated with CS and Alg. CCNZ&Alg, with a negative charge, can specifically bind to intestinal inflammatory sites with a positive charge, optimizing the ratio of CCNZ to Alg for better adhesion and antibacterial performance. ROS-sensitive fluorescent probe results demonstrated that CCNZ_1_:Alg_1.5_ effectively reduces ROS levels, thereby alleviating the inflammatory response associated with IBD [59]. In another study, dextran-coated cerium oxide (D-CeO_2_) nanoenzymes were formed by chemical precipitation, which were encapsulated in CS/Alg brine gel to effectively remove ROS. Meanwhile, immunofluorescence showed that D-CeO_2_ had good fiber resistance. Masson staining showed that no fibrin deposition was observed under colonic mucosa in the TNBS + D-CeO_2_ group [60].

### 4.4. Growth Factors

Poloxamer 407 can be developed as a temperature-sensitive hydrogel that transitions into a semi-solid state at body temperature when present at concentrations of 17% or higher, allowing for diffusion within the colon wall [61]. Moreover, the hydrogel prepared from epigallocatechin-3-gallate (EGCG) extracted from green tea has good antioxidant and healing effects [62,63]. Therefore, poloxamer 407 is used to form a polymer skeleton, EGCG is used to enhance the adhesion of the hydrogel, an adhesive in situ thermosensitive hydrogel (PE) is developed, and keratinocyte growth factor (KGF) is encapsulated in the PE hydrogel to form KGF@PE hydrogel, which can promote the obvious migration of NIH3T3 cells and promote the healing of the intestinal epithelial barrier. After rectal administration, EGCG can prolong the retention time of KGF@PE in the colon and promote the production of IL-10 [64].

Previously, a thermosensitive hydrogel of dihydrocaffeic acid-modified poloxamer (DAP) was developed, which exhibited good mechanical properties and strong anti-eroding effects. However, it could not achieve specific adhesion to colitis mucosa [65]. DAP was combined with EGCG and HA to form an in situ hydrogel (PHE), and subsequently the growth factors KPV and EGF were loaded into the PHE hydrogel, resulting in a mucin-inspired hydrogel (PHE-EK) with adhesive properties. This hydrogel can rapidly gel at body temperature and specifically adhere to the inflamed colon through electrostatic interactions, reducing the infiltration of inflammatory cells and alleviating the degree of inflammation [66].

### 4.5. Probiotics

Although the exact etiology of UC remains under debate, the intestinal flora play an undeniable and crucial role in the pathogenesis of UC [67]. *Lactobacillus rhamnosus* GG (LGG) in lactic acid bacteria can compete for binding sites with pathogens, thereby inhibiting their adhesion and colonization on intestinal epithelium. LGG was encapsulated in a mercaptan-modified HA hydrogel to form a HA-SH hydrogel, enhance colon targeting, and improve the inflammation degree in a *Salmonella typhimurium* (ST)-induced colitis model [68]. In another study, an ROS-responsive hydrogel (HA-LR) loaded with *Lactobacillu reuteri* (LR) demonstrated an approximately 90% survival rate of LR. Immunofluorescence revealed that post H_2_O_2_ treatment of RAW264.7 cells to establish a cellular inflammatory model, HA-LR treatment markedly reduced ROS levels, attenuated oxidative stress, and alleviated colitis severity [69].

The cytokines, such as TNF-α and IL-1β, can modulate immune responses in the intestinal epithelium, leading to the development of monoclonal antibodies targeting these cytokines for patients who do not respond to conventional treatments [70,71]. The conjugation of GPM-EcN not only alleviates the M1 polarization induced by EcN and significantly suppresses the up-regulation of inflammatory cytokines such as TNF-α and IL-1β, but also protects Caco-2 cells and restores the gut epithelial monolayer barrier [33]. In another recent study, macrophages were co-cultured with 10% Poly ethylene glycol diacrylate and 1% I2959. After freeze-thaw cycles (−80 °C freezing and room-temperature thawing), the hydrogel material permeated into the macrophage cytoplasm and formed intracellular gelated macrophages (GMs) upon UV irradiation. EcN was loaded into GM via phagocytosis to form GM-EcN. GM could bind to inflammatory factors through Tumor Necrosis Factor Receptor 2 and interleukin-1 receptor type 2 on the membrane to reduce inflammation [72].

In addition, the Alg gel was used to encapsulate bifidobacterium (Bac) and drug-modified nanoscale dietary fibers (NDFs) through the electrostatic droplet generator to create hydrogel microspheres (NDF-Ms), which led to a significant reduction in CD11b^+^Ly6G^+^ neutrophils and CD11b^+^F4/80^+^ macrophages, resulting in a marked decrease in the inflammatory response of 5-ASA in NDF-M1/H at the site of intestinal inflammation [32].

### 4.6. Liposome Complexes

Liposomes can avoid the first-pass effect, and have advantages such as good biocompatibility, low immunogenicity, and low toxicity [73]. By designing Bud _Lip-Gel_, the solubility of Bud can be improved, which helps to sustain its release. After rectal administration of Bud _Lip-Gel_, compared to the Bud group, the Disease Activity Index score was lower, and the expression level of the pro-inflammatory cytokine IL-6 in serum was down-regulated [74]. Previously, it was reported that modified liposomes were loaded with CUR with folic acid (FA) to form macrophage-targeted liposomes (FA/CUR PEG LPs), but these liposomes are unstable in acidic environments and prone to premature release [75]. To address this issue, FA/CUR-PEG-LP was wrapped with PC hydrogels, and the hydrogel component enhanced the stability of liposomes in SIF. Immunofluorescence results demonstrated that FA/CUR-PEG-LPs@PC possesses significant targeting ability towards colonic macrophages, thereby improving the efficacy of treatment for UC [76].

### 4.7. Plant Compounds

Plant compounds such as Cannabidiol (CBD), Que, etc., can exhibit good anti-inflammatory and antioxidant properties, polarize M2 macrophages, reduce the production of pro-inflammatory cytokines, and inhibit oxidative stress [77]. For example, CBD loaded into GelMA not only preserves the structural integrity of GelMA but also enhances the adhesion, proliferation, and migration of RAW264.7 cells. Furthermore, GelMA + CBD can significantly inhibit oxidative stress, up-regulate the expression of M2-associated proteins in macrophages, and suppress the expression of inducible nitric oxide synthase [78]. Que, demethyleneberberine, and dencichine were co-encapsulated in a guanosine-based hydrogel to construct a multi-drug-loaded hydrogel co-delivery system (GB/D_3_ hydrogel) through the combination of borate ester bonds and the reaction of guanosine borate. After injection administration, the encapsulated drugs are released in an environment of weak acid and excessive ROS, reducing the levels of pro-inflammatory cytokines such as TNF-α and IL-6, which is beneficial for colitis repair [79].

### 4.8. Proteins and Peptides

A CS/Alg hydrogel containing silk sericin can improve the selective permeation of silk sericin in the intestine, and significantly increase the expression levels of anti-inflammatory cytokines and TJs (ZO-1 and occludin) [80]. As another paradigm, the self-crosslinking SH-PGA hydrogel was formed by conjugating cysteamine hydrochloride with the carboxyl group of γ-PGA to load KPV (KPV/SH-PGA hydrogel), and the healing rate of the wound was twice as high as that of the control group [81]. Subsequently, the research team modified γ-polyglutamic acid by maleimide amination and thiolation to form a double-network hydrogel, which can regulate the abundance of relevant intestinal flora, such as reducing the number of *Proteobacteria* and *Verrucomicrobia*, and increasing the number of *Firmicutes* [82]. Hyo-Jin Yoon et al. sprayed a peptide(Pe)-hydrogel onto the porcine colon wall during colonoscopy, achieving stable diffusion of the hydrogel on the colon wall. The Pe-hydrogel can bind to TLR5 through Pe guidance, significantly reducing the expression of pro-inflammatory cytokines and inhibiting the NF-κB signaling pathway to suppress inflammation [83].

### 4.9. Nanoparticles (NPs)

NPs could enhance tissue targeting and reduce systemic toxicity caused by long-term or frequent administration of conventional drugs through strategies such as altering particle size, charge modification, surface modification with specific antibodies, and coating with a cell membrane layer [84].

TNFα siRNA could be effectively loaded into NPs made from -poly (lactic acid) poly (ethylene glycol) block copolymer (PLA-PEG), and the surface of these NPs was modified with Fab’ to enhance targeting to macrophages. Alg and CS (7:3) were used to form hydrogels to encapsulate and protect the NPs, and inhibit the NF-κB signaling pathway via the inhibitor of κBα protein, thereby reducing inflammation in DSS mice [85]. Platinum NPs (Pt NPs), as a novel class of nanomaterials with superoxide dismutase and catalase activities, can exert antioxidant effects in liver ischemia-reperfusion injury [86]. CS/Alg hydrogels were used to encapsulate Pt NPs, enhancing their targeting to the intestine and protecting the Pt NPs from gastric acid damage, thereby improving their anti-inflammatory and antioxidant abilities [87]. A dual-targeted drug delivery system (HA-PLGA_bilirubin_) was developed by surface-modifying poly (lactic-co-glycolic acid) NPs with negatively charged HA and encapsulating bilirubin within a CS/Alg hydrogel delivery system, which demonstrated a significantly lower bilirubin release rate in simulated colonic fluid compared to simulated gastric fluid. Moreover, the HA-modified poly (lactic-co-glycolic acid) NPs could bind to CD44 ligands at colonic inflammation sites to enable dual targeting of the colon, therefore markedly increasing the proliferation of Lgr5^+^ intestinal epithelial stem cells in mice [88].

6-Shogaol, known for its antioxidant, anti-inflammatory, and anti-cancer properties, can be specifically delivered to colon inflammation sites by folate-functionalized 6-shogaol-loaded PLGA (Lactide:glycolide (75:25))/PLA NPs encapsulated in a CS/Alg hydrogel delivery system, as folate targets the folate receptors expressed in intestinal epithelial cells, and the results demonstrated that oral administration of NPs-PEG-FA/6-shogaol to DSS-treated mice significantly reduced lipocalin-2 expression, indicating remarkable anti-inflammatory effects [89].

## 5. Current Problems and Next Research Directions

Hydrogels, due to their unique properties, such as their excellent protective properties, mechanical strength, injectability, and sensitivity to stimuli, have gained widespread attention in the field of biomedicine, particularly as incomparable drug delivery carriers [8]. However, whether different types of hydrogels can maintain the bioactivity of drugs and whether they affect cells and tissues is difficult to control and predict. Although hydrogel delivery systems can deliver drugs, exert anti-inflammatory effects, and repair the gut barrier in IBD treatment, these systems cannot precisely deliver materials to the target site, and their drug sustained-release effect and duration of action are unstable, which impairs IBD treatment [6,30]. For instance, though rectal administration of the thermosensitive Gel-Tel hydrogel can achieve therapeutic effects, it is hard to precisely control the gelation site in the intestine [31]. In addition, SC-HM can greatly decrease the mRNA expression of Toll-like receptors (TLR-4, -5, and -9) linked to the gut microbiota, but it does not fundamentally resolve inflammation and dysbiosis, needing further in-depth exploration [16]. Thus, high-precision hydrogel delivery systems should be developed to achieve accurate control over drug release.

Macrophages and stem cells both play crucial roles in the treatment of IBD. Hydrogel binding with hP-MSCs promotes PGE_2_ secretion and M2 polarization of macrophages [47]. However, the mechanism of how PGE_2_ regulates the specific signal pathway of macrophages is not thoroughly studied, and whether hydrogels have other effects on stem cells is still unknown. In addition, short-term efficacy is not sufficient to evaluate long-term efficacy, so we should try to extend the observation time as much as possible.

Currently, animal models for IBD-related studies are mainly based on trinitrobenzene sulfonic acid [65] and DSS [90] models in rats [91] or mice [11], lacking large animal models and long-term experimental data. Furthermore, hydrogel delivery systems still face several challenges to achieve clinical translation. While the PC/PAM hydrogel has shown promising slow-release properties through in vitro experiments, it has not been further validated histologically, and its biosafety and stability need to be confirmed by further studies [14]. The Pe-hydrogel, despite being tested in pigs, has an inadequate sample size [83]. Additionally, given the annual global increase in IBD patients, the clinical-scale application of hydrogels as a delivery system and their production costs must be considered. Some hydrogels, such as HA-PLGA_bilirubin_ [88] and SC-HM [16], have complex production processes, stringent storage and transportation requirements, and high costs, which hinder their clinical-scale production and application. Overall, the hydrogel delivery system requires further optimization to promote its transfer from lab scale to clinical scale. 

## 6. Conclusions

In recent years, hydrogels have developed from single drug carriers to smart responsive hydrogels capable of drug delivery, anti-inflammation effects, and repair of the intestinal barrier, which are widely used in the medical field. This review explores the innovative applications of hydrogel-based drug-delivery strategies, such as ROS/pH dual-responsive hydrogels and self-assembled hydrogels. While hydrogels show great potential as carriers for efficient drug delivery and avoiding gastrointestinal pH and enzymatic degradation, developing more effective drug-delivery methods is still crucial. Modified hydrogels, as delivery systems, can enhance intestinal mucosa adhesion, which may affect intraluminal material dissolution. Therefore, further research to optimize the application of these systems is required.

## Figures and Tables

**Table 1:** Drug delivery modes and sustained-release effects of hydrogels.

Hydrogel Name	Response Mode	Delivery Method	Delivery of Substances	Application Forms of Hydrogel	Sustained-Release Effect	References
GBR hydrogel	ROS/pH dual-responsive hydrogel	Rectal injection administration	Rutin	Injectable hydrogel	pH = 7.4, 37 °C, 24 h, the release rate is less than 35%, and 20% at 25 °C; pH = 6.8, 37 °C, 12 h, the release rate is approximately 70% and 85% after 24 h; pH = 6.8 + 10 μM H_2_O_2_, 37 °C, 8 h, the release rate exceeds 90%, and nearly complete release occurs after 12 h, with a faster release rate.	[10]
HEP-Ag-BSA	Versatile protein hydrogel	Rectal enema administration	HEP	Injectable hydrogel	1 d, the heparin release rate was 40.4 ± 4.51%; 8 d, the release rate increased to 93.3 ± 4.16%; 1 d, Ag^+^ release rate was 44.7 ± 4.46%; 8 d, the release rate increased to 94.7 ± 3.06%.	[11]
C/S-MSe	pH-sensitive hydrogel	Oral administration	M-SeNPs	Hydrogel bead	pH = 1.2, 24 h, the cumulative release of M-SeNPs from C/S-MSe hydrogel microbeads is 19.8%; pH = 6.8, 24 h, the cumulative release of M-SeNPs from C/S-MSe hydrogel microbeads is 46.1%.	[12]
Cu_2_(Olsa)/Gel	pH-sensitive hydrogel	Oral administration	Cu_2_(Olsa) nanoneedle	bioMOF/hydrogel platform	In SIF, 4 h, the release of Olsalazine sodium is 71.7%; pH = 5–6, the Olsa release rate is 20.3%.	[13]
PC/PAM hydrogel	pH-sensitive enzyme-triggered hydrogel	Rectal administration	Bud	Hydrogel-based composite drug delivery system	PC/PAM hydrogel under the F3 optimized formulation: pH = 1.5, 2 h, the cumulative release rate is 7%; pH = 7.4, 3 h, the cumulative release rate is 36%.	[14]

**Table 2:** Application forms and sustained-release effects of hydrogels.

Hydrogel Name	Response Mode	Delivery Method	Delivery of Substances	Sustained-Release Effect	References
CUR/EMO NE@SA	pH-sensitive hydrogel	Oral administration	CUR and EMO	pH = 1.2, 2 h, little drug release; pH = 6.8, 4 h, the cumulative drug release was about 20%; pH = 7.8, the cumulative drug release was about 70%.	[23]
SP@Rh-Gel	pH-sensitive hydrogel	Oral administration	Rh	pH = 1.8, the Rh release rate is minimal; pH = 7.4, 72 h, the Rh release rate reaches 100%.	[24]
EcN@Gel	NTR-labile peptidic hydrogel	Oral administration	EcN	pH = 2.5, NTR is absent, 2 h, EcN releases almost none; pH = 7.8, NTR is present, 6 h, EcN exhibits the fastest and largest release; PBS (pH = 6.8) + NTR releases more NTR than PBS (pH = 7.8).	[25]
PPy/PFD@lnulin gel	pH-sensitive hydrogel	Oral administration	PPy and PFD	Prolonged and complete release within 24 h.	[26]
Pur@HA-SH-zein NPs in hydrogel	Thermosensitive hydrogel	Rectal injection administration	Puerarin	48 h, the cumulative release rate was 77.61%.	[27]
GBQ hydrogel	ROS/pH dual-responsive hydrogel	Rectal enema administration	Que	pH = 6.8, 8 h, the release rate was 82.7%; 24 h, it reached 92%; Add H_2_O_2_, 8 h, the release rate increased to 95.2%; 24 h, it reached 99%. pH = 7.4, 8 h, the release rate was 65.4%; 24 h, it was 78%.	[28]
HCD-bFGF-ALG Hydrogel	Multifunctional mechanically- resilient self-healing hydrogel	Rectal enema administration	DEX, bFGF, ALG	The amount of residual hydrogel is 15% at pH 5.5 and 28% at pH 7.4.	[29]
Mesa/Gel	Injectable thermosensitive hydrogel	Rectal injection administration	Me	In PBS at 37 °C, the cumulative release rates of Mesa in Mesa/P2 and Mesa/P4 are 79.3% and 76.1%, respectively.	[30]
Gel-Tel	Thermosensitive hydrogel	Rectal injection administration	Tel	In simulated colonic fluid, after 12 h, Gel-Tel achieved only approximately a 50% release rate.	[31]

## Data Availability

No new data were created or analyzed in this study.

## References

[1] Stemmer E., Zahavi T., Kellerman M., Sinberger L.A., Shrem G., Salmon-Divon M. (2024). Exploring potential biomarkers and therapeutic targets in inflammatory bowel disease: Insights from a mega-analysis approach. Front. Immunol..

[2] Roda G., Chien Ng S., Kotze P.G., Argollo M., Panaccione R., Spinelli A., Kaser A., Peyrin-Biroulet L., Danese S. (2020). Crohn’s disease. Nat. Rev. Dis. Primers.

[3] Larabi A., Barnich N., Nguyen H.T.T. (2020). New insights into the interplay between autophagy, gut microbiota and inflammatory responses in IBD. Autophagy.

[4] Cao F., Jin L., Gao Y., Ding Y., Wen H., Qian Z., Zhang C., Hong L., Yang H., Zhang J. (2023). Artificial-enzymes-armed bifidobacterium longum probiotics for alleviating intestinal inflammation and microbiota dysbiosis. Nat. Nanotechnol..

[5] Gao M., Yang C., Wu C., Chen Y., Zhuang H., Wang J., Cao Z. (2022). Hydrogel-metal-organic-framework hybrids mediated efficient oral delivery of sirna for the treatment of ulcerative colitis. J. Nanobiotechnology.

[6] Sohail M., Mudassir, Minhas M.U., Khan S., Hussain Z., De Matas M., Shah S.A., Khan S., Kousar M., Ullah K. (2019). Natural and synthetic polymer-based smart biomaterials for management of ulcerative colitis: A review of recent developments and future prospects. Drug Deliv. Transl. Res..

[7] Xiao C., Li G., Li X., Wang D., Wu Y., Sun M., Huang J., Si L. (2023). A topical thermosensitive hydrogel system with cyclosporine A PEG-PCL micelles alleviates ulcerative colitis induced by TNBS in mice. Drug Deliv. Transl. Res..

[8] Cao H., Duan L., Zhang Y., Cao J., Zhang K. (2021). Current hydrogel advances in physicochemical and biological response-driven biomedical application diversity. Signal Transduct. Target. Ther..

[9] Pal A., Vernon B.L., Nikkhah M. (2018). Therapeutic neovascularization promoted by injectable hydrogels. Bioact. Mater..

[10] Wang H., Wang L., Guo S., Liu Z., Zhao L., Qiao R., Li C. (2022). Rutin-loaded stimuli-responsive hydrogel for anti-inflammation. ACS Appl. Mater. Interfaces.

[11] Hong L., Chen G., Cai Z., Liu H., Zhang C., Wang F., Xiao Z., Zhong J., Wang L., Wang Z. (2022). Balancing microthrombosis and inflammation via injectable protein hydrogel for inflammatory bowel disease. Adv. Sci..

[12] Yang H., Wang Z., Li L., Wang X., Wei X., Gou S., Ding Z., Cai Z., Ling Q., Hoffmann P.R. (2024). Mannose coated selenium nanoparticles normalize intestinal homeostasis in mice and mitigate colitis by inhibiting NF-κB activation and enhancing glutathione peroxidase expression. J. Nanobiotechnol..

[13] Zhang Z., Pan Y., Guo Z., Fan X., Pan Q., Gao W., Luo K., Pu Y., He B. (2024). An olsalazine nanoneedle-embedded inulin hydrogel reshapes intestinal homeostasis in inflammatory bowel disease. Bioact. Mater..

[14] Pandey M., Choudhury H., D/O Segar Singh S.K., Chetty Annan N., Bhattamisra S.K., Gorain B., Mohd Amin M.C.I. (2021). Budesonide-loaded pectin/polyacrylamide hydrogel for sustained delivery: Fabrication, characterization and in vitro release kinetics. Molecules.

[15] Chen T., Chen L., Luo F., Xu Y., Wu D., Li Y., Zhao R., Hua Z., Hu J. (2023). Efficient oral delivery of resveratrol-loaded cyclodextrin-metal organic framework for alleviation of ulcerative colitis. Int. J. Pharm..

[16] Kim D.-W., Jeong H.-S., Kim E., Lee H., Choi C.-H., Lee S.-J. (2022). Oral delivery of stem-cell-loaded hydrogel microcapsules restores gut inflammation and microbiota. J. Control. Release.

[17] Huang L., Mao X., Li Y., Liu D., Fan K., Liu R., Wu T., Wang H., Zhang Y., Yang B. (2021). Multiomics analyses reveal a critical role of selenium in controlling t cell differentiation in Crohn’s disease. Immunity.

[18] Torretta S., Scagliola A., Ricci L., Mainini F., Di Marco S., Cuccovillo I., Kajaste-Rudnitski A., Sumpton D., Ryan K.M., Cardaci S. (2020). D-mannose suppresses macrophage IL-1Β production. Nat. Commun..

[19] Zhang S., Kang L., Hu S., Hu J., Fu Y., Hu Y., Yang X. (2021). Carboxymethyl chitosan microspheres loaded hyaluronic acid/gelatin hydrogels for controlled drug delivery and the treatment of inflammatory bowel disease. Int. J. Biol. Macromol..

[20] Xu S., Hu B., Dong T., Chen B., Xiong X., Du L., Li Y., Chen Y., Tian G., Bai X. (2023). Alleviate periodontitis and its comorbidity hypertension using a nanoparticle-embedded functional hydrogel system. Adv. Healthc. Mater..

[21] Han K., Nam J., Xu J., Sun X., Huang X., Animasahun O., Achreja A., Jeon J.H., Pursley B., Kamada N. (2021). Generation of systemic antitumour immunity via the in situ modulation of the gut microbiome by an orally administered inulin gel. Nat. Biomed. Eng..

[22] Yu H., Gao R., Liu Y., Fu L., Zhou J., Li L. (2024). Stimulus-responsive hydrogels as drug delivery systems for inflammation targeted therapy. Adv. Sci..

[23] Lei F., Zeng F., Yu X., Deng Y., Zhang Z., Xu M., Ding N., Tian J., Li C. (2023). Oral hydrogel nanoemulsion co-delivery system treats inflammatory bowel disease via anti-inflammatory and promoting intestinal mucosa repair. J. Nanobiotechnol..

[24] Zhong D., Jin K., Wang R., Chen B., Zhang J., Ren C., Chen X., Lu J., Zhou M. (2024). Microalgae-based hydrogel for inflammatory bowel disease and its associated anxiety and depression. Adv. Mater..

[25] Chen J., Zhang P., Wu C., Yao Q., Cha R., Gao Y. (2023). Reductase-labile peptidic supramolecular hydrogels aided in oral delivery of probiotics. ACS Appl. Mater. Interface.

[26] Cao X., Tao S., Wang W., Wu S., Hong Y., Wang X., Ma Y., Qian H., Zha Z. (2024). Ternary inulin hydrogel with long-term intestinal retention for simultaneously reversing IBD and its fibrotic complication. Nat. Commun..

[27] Zhao S., Zhang J., Qiu M., Hou Y., Li X., Zhong G., Gou K., Li J., Zhang C., Qu Y. (2024). Mucoadhesive and thermosensitive bletilla striata polysaccharide/chitosan hydrogel loaded nanoparticles for rectal drug delivery in ulcerative colitis. Int. J. Biol. Macromol..

[28] Zhao L., Dou D., Zhang D., Deng X., Ding N., Ma Y., Ji X., Zhang S., Li C. (2024). ROS/pH dual-responsive quercetin-loaded guanosine borate supramolecular hydrogel enema in dextran sulfate sodium-induced colitis in mice. J. Mater. Chem. B.

[29] Ge X., Wen H., Fei Y., Xue R., Cheng Z., Li Y., Cai K., Li L., Li M., Luo Z. (2023). Structurally dynamic self-healable hydrogel cooperatively inhibits intestinal inflammation and promotes mucosal repair for enhanced ulcerative colitis treatment. Biomaterials.

[30] Guo Z., Bai Y., Zhang Z., Mei H., Li J., Pu Y., Zhao N., Gao W., Wu F., He B. (2021). Thermosensitive polymer hydrogel as a physical shield on colonic mucosa for colitis treatment. J. Mater. Chem. B.

[31] Xu L., Zhao Q., Xie Y., Bai G., Liu H., Chen Q., Duan H., Wang L., Xu H., Sun Y. (2024). Telmisartan loading thermosensitive hydrogel repairs gut epithelial barrier for alleviating inflammatory bowel disease. Colloids Surf. B Biointerfaces.

[32] Qiu L., Shen R., Wei L., Xu S., Xia W., Hou Y., Cui J., Qu R., Luo J., Cao J. (2023). Designing a microbial fermentation-functionalized alginate microsphere for targeted release of 5-ASA using nano dietary fiber carrier for inflammatory bowel disease treatment. J. Nanobiotechnol..

[33] Ren X., Chen D., Wang Y., Li H., Zhang Y., Chen H., Li X., Huo M. (2022). Nanozymes-recent development and biomedical applications. J. Nanobiotechnology.

[34] Bisgaard T.H., Allin K.H., Keefer L., Ananthakrishnan A.N., Jess T. (2022). Depression and anxiety in inflammatory bowel disease: Epidemiology, mechanisms and treatment. Nat. Rev. Gastroenterol. Hepatol..

[35] Liu J., Wang Y., Heelan W.J., Chen Y., Li Z., Hu Q. (2022). Mucoadhesive probiotic backpacks with ros nanoscavengers enhance the bacteriotherapy for inflammatory bowel diseases. Sci. Adv..

[36] Koh E., Hwang I.Y., Lee H.L., De Sotto R., Lee J.W.J., Lee Y.S., March J.C., Chang M.W. (2022). Engineering probiotics to inhibit clostridioides difficile infection by dynamic regulation of intestinal metabolism. Nat. Commun..

[37] Zhang X., Zhu Y., Xiong Z., Xie W., Shao M., Liu Z. (2025). Broad-spectrum ROS/RNS scavenging catalase-loaded microreactors for effective oral treatment of inflammatory bowel diseases. Small.

[38] Wan J., Yu X., Liu J., Li J., Ai T., Yin C., Liu H., Qin R. (2023). A special polysaccharide hydrogel coated on *Brasenia schreberi*: Preventive effects against ulcerative colitis *via* modulation of gut microbiota. Food. Funct..

[39] Wang J., Hu D., Chen Q., Liu T., Zhou X., Xu Y., Zhou H., Gu D., Gao C. (2023). Intracellular hydrogelation of macrophage conjugated probiotics for hitchhiking delivery and combined treatment of colitis. Mater. Today Bio..

[40] Bialik M., Kuras M., Sobczak M., Oledzka E. (2021). Achievements in thermosensitive gelling systems for rectal administration. Int. J. Mol. Sci..

[41] Xu J., Tam M., Samaei S., Lerouge S., Barralet J., Stevenson M.M., Cerruti M. (2017). Mucoadhesive chitosan hydrogels as rectal drug delivery vessels to treat ulcerative colitis. Acta. Biomater..

[42] Biamonte P., D’Amico F., Fasulo E., Barà R., Bernardi F., Allocca M., Zilli A., Danese S., Furfaro F. (2023). New technologies in digestive endoscopy for ulcerative colitis patients. Biomedicines.

[43] Kou Y., Li J., Zhu Y., Liu J., Ren R., Jiang Y., Wang Y., Qiu C., Zhou J., Yang Z. (2024). Human amniotic epithelial stem cells promote colonic recovery in experimental colitis via exosomal MiR-23a-TNFR1-NF-κB signaling. Adv. Sci..

[44] Nagai K., Ideguchi H., Kajikawa T., Li X., Chavakis T., Cheng J., Messersmith P.B., Heber-Katz E., Hajishengallis G. (2020). An injectable hydrogel-formulated inhibitor of prolyl-4-hydroxylase promotes t regulatory cell recruitment and enhances alveolar bone regeneration during resolution of experimental periodontitis. FASEB J..

[45] DeFrates K.G., Tong E., Cheng J., Heber-Katz E., Messersmith P.B. (2024). A pro-regenerative supramolecular prodrug protects against and repairs colon damage in experimental colitis. Adv. Sci..

[46] Gonzalez-Pujana A., Beloqui A., Javier Aguirre J., Igartua M., Santos-Vizcaino E., Maria Hernandez R. (2022). Mesenchymal stromal cells encapsulated in licensing hydrogels exert delocalized systemic protection against ulcerative colitis via subcutaneous xenotransplantation. Eur. J. Pharm. Biopharm..

[47] Cao X., Duan L., Hou H., Liu Y., Chen S., Zhang S., Liu Y., Wang C., Qi X., Liu N. (2020). IGF-1C hydrogel improves the therapeutic effects of MSCs on colitis in mice through PGE_2_-mediated M2 macrophage polarization. Theranostics.

[48] Mehta R.S., Mayers J.R., Zhang Y., Bhosle A., Glasser N.R., Nguyen L.H., Ma W., Bae S., Branck T., Song K. (2023). Gut microbial metabolism of 5-ASA diminishes its clinical efficacy in inflammatory bowel disease. Nat. Med..

[49] Fu Z., Zou J., Zhong J., Zhan J., Zhang L., Xie X., Zhang L., Li W., He R. (2024). Curcumin-loaded nanocomposite hydrogel dressings for promoting infected wound healing and tissue regeneration. Int. J. Nanomed..

[50] Zeng Y.-X., Wang S., Wei L., Cui Y.-Y., Chen Y.-H. (2020). Proanthocyanidins: Components, pharmacokinetics and biomedical properties. Am. J. Chin. Med..

[51] Kuo S.-N., Wu P.-X., Huang S.-L., Hsu Y.-C., Huang J.-H. (2025). Thermo-responsive methylcellulose/hyaluronic acid-mesalamine hydrogel in targeted drug delivery for ulcerative colitis. RSC Adv..

[52] Lan T., Luo M., Wei X. (2021). Mesenchymal stem/stromal cells in cancer therapy. J. Hematol. Oncol..

[53] Riazifar M., Mohammadi M.R., Pone E.J., Yeri A., Lässer C., Segaliny A.I., McIntyre L.L., Shelke G.V., Hutchins E., Hamamoto A. (2019). Stem cell-derived exosomes as nanotherapeutics for autoimmune and neurodegenerative disorders. ACS Nano.

[54] Xiao K., He W., Guan W., Hou F., Yan P., Xu J., Zhou T., Liu Y., Xie L. (2020). Mesenchymal stem cells reverse EMT process through blocking the activation of NF-κB and Hedgehog pathways in LPS-induced acute lung injury. Cell Death Dis..

[55] Khayambashi P., Iyer J., Pillai S., Upadhyay A., Zhang Y., Tran S. (2021). Hydrogel encapsulation of mesenchymal stem cells and their derived exosomes for tissue engineering. Int. J. Mol. Sci..

[56] Nie M., Huang D., Chen G., Zhao Y., Sun L. (2023). Bioadhesive microcarriers encapsulated with IL-27 high expressive msc extracellular vesicles for inflammatory bowel disease treatment. Adv. Sci..

[57] De Vos W.M., Tilg H., Van Hul M., Cani P.D. (2022). Gut microbiome and health: Mechanistic insights. Gut.

[58] Yu Y., Zhao S., Gu D., Zhu B., Liu H., Wu W., Wu J., Wei H., Miao L. (2022). Cerium oxide nanozyme attenuates periodontal bone destruction by inhibiting the ROS-NFκB pathway. Nanoscale.

[59] Cheng C., Cheng Y., Zhao S., Wang Q., Li S., Chen X., Yang X., Wei H. (2022). Multifunctional nanozyme hydrogel with mucosal healing activity for single-dose ulcerative colitis therapy. Bioconjug. Chem..

[60] Cao Y., Cheng K., Yang M., Deng Z., Ma Y., Yan X., Zhang Y., Jia Z., Wang J., Tu K. (2023). Orally administration of cerium oxide nanozyme for computed tomography imaging and anti-inflammatory/anti-fibrotic therapy of inflammatory bowel disease. J. Nanobiotechnology.

[61] Xue P., Wang L., Xu J., Liu J., Pan X., Zhao Y., Xu H. (2020). Temperature-sensitive hydrogel for rectal perfusion improved the therapeutic effect of kangfuxin liquid on DSS-induced ulcerative colitis mice: The inflammation alleviation and the colonic mucosal barriers repair. Int. J. Pharm..

[62] Wu Z., Huang S., Li T., Li N., Han D., Zhang B., Xu Z.Z., Zhang S., Pang J., Wang S. (2021). Gut microbiota from green tea polyphenol-dosed mice improves intestinal epithelial homeostasis and ameliorates experimental colitis. Microbiome.

[63] Yongvongsoontorn N., Chung J.E., Gao S.J., Bae K.H., Yamashita A., Tan M.-H., Ying J.Y., Kurisawa M. (2019). Carrier-enhanced anticancer efficacy of sunitinib-loaded green tea-based micellar nanocomplex beyond tumor-targeted delivery. ACS Nano.

[64] Lin G., Yang J., Liu J., Shangguan J., Pan H., Zhang Y., Ran K., Li D., Yu F., Xu H. (2023). In situ polyphenol-adhesive hydrogel enhanced the noncarcinogenic repairing of KGF on the gut epithelial barrier on TNBS-induced colitis rats. Int. J. Biol. Macromol..

[65] Wang L., Xu J., Xue P., Liu J., Luo L., Zhuge D., Yao Q., Li X., Zhao Y., Xu H. (2021). Thermo-sensitive hydrogel with mussel-inspired adhesion enhanced the non-fibrotic repair effect of EGF on colonic mucosa barrier of TNBS-induced ulcerative colitis rats through macrophage polarizing. Chem. Eng. J..

[66] Li D., Shangguan J., Yu F., Lin G., Pan H., Zhang M., Lin H., Chen B., Xu H., Hu S. (2024). Growth factors-loaded temperature-sensitive hydrogel as biomimetic mucus attenuated murine ulcerative colitis via repairing the mucosal barriers. ACS Appl. Mater. Interfaces.

[67] Lee J.-Y., Tsolis R.M., Bäumler A.J. (2022). The microbiome and gut homeostasis. Science..

[68] Xiao Y., Lu C., Liu Y., Kong L., Bai H., Mu H., Li Z., Geng H., Duan J. (2020). Encapsulation of *Lactobacillus rhamnosus* in hyaluronic acid-based hydrogel for pathogen-targeted delivery to ameliorate enteritis. ACS Appl. Mater. Interfaces.

[69] Huang L., Wang J., Kong L., Wang X., Li Q., Zhang L., Shi J., Duan J., Mu H. (2022). ROS-responsive hyaluronic acid hydrogel for targeted delivery of probiotics to relieve colitis. Int. J. Biol. Macromol..

[70] Mahapatro M., Erkert L., Becker C. (2021). Cytokine-mediated crosstalk between immune cells and epithelial cells in the gut. Cells.

[71] Stallmach A., Atreya R., Grunert P.C., Stallhofer J., De Laffolie J., Schmidt C. (2023). Treatment Strategies in Inflammatory Bowel Diseases. Dtsch. Arztebl. Int..

[72] Gu S., Zhao X., Wan F., Gu D., Xie W., Gao C. (2024). Intracellularly gelated macrophages loaded with probiotics for therapy of colitis. Nano Lett..

[73] Li M., Du C., Guo N., Teng Y., Meng X., Sun H., Li S., Yu P., Galons H. (2019). Composition design and medical application of liposomes. Eur. J. Med. Chem..

[74] Yang D., Zhao Y., Teng L., He J., Zhao J., Gao X., Ma G. (2023). Budesonide-liposomes inclusion complex in thermosensitive gel alleviates DSS-induced ulcerative colitis in mice. Drug Dev. Ind. Pharm..

[75] Yuba E. (2020). Development of functional liposomes by modification of stimuli-responsive materials and their biomedical applications. J. Mater. Chem. B.

[76] Wu M., Ping H., Wang K., Ding H., Zhang M., Yang Z., Du Q. (2023). Oral delivery of pectin-chitosan hydrogels entrapping macrophage-targeted curcumin-loaded liposomes for the treatment of ulcerative colitis. Int. J. Pharm..

[77] Silvestri C., Pagano E., Lacroix S., Venneri T., Cristiano C., Calignano A., Parisi O.A., Izzo A.A., Di Marzo V., Borrelli F. (2020). Fish oil, cannabidiol and the gut microbiota: An investigation in a murine model of colitis. Front. Pharmacol..

[78] Wang Y., Ji X., Wang X., Sun M., Li C., Wu D. (2024). The injectable hydrogel loading cannabidiol to regulate macrophage polarization in vitro for the treatment of chronic enteritis. J. Appl. Biomater. Funct. Mater..

[79] Gao W., Zhang D., Wang H., Qiao R., Li C. (2024). Guanosine-based multidrug strategy delivery for synergistic anti-inflammation. ACS Macro. Lett..

[80] Ma Y., Tong X., Huang Y., Zhou X., Yang C., Chen J., Dai F., Xiao B. (2019). Oral administration of hydrogel-embedding silk sericin alleviates ulcerative colitis through wound healing, anti-inflammation, and anti-oxidation. ACS Biomater. Sci. Eng..

[81] Sun J., Xue P., Liu J., Huang L., Lin G., Ran K., Yang J., Lu C., Zhao Y.-Z., Xu H.-L. (2021). Self-cross-linked hydrogel of cysteamine-grafted γ-polyglutamic acid stabilized tripeptide KPV for alleviating TNBS-induced ulcerative colitis in rats. ACS Biomater. Sci. Eng..

[82] Zhao Y., Xue P., Lin G., Tong M., Yang J., Zhang Y., Ran K., Zhuge D., Yao Q., Xu H. (2022). A KPV-binding double-network hydrogel restores gut mucosal barrier in an inflamed colon. Acta Biomater..

[83] Yoon H.-J., Lee S., Kim T.Y., Yu S.E., Kim H.-S., Chung Y.S., Chung S., Park S., Shin Y.C., Wang E.K. (2022). Sprayable nanomicelle hydrogels and inflammatory bowel disease patient cell chips for development of intestinal lesion-specific therapy. Bioact. Mater..

[84] Brusini R., Varna M., Couvreur P. (2020). Advanced nanomedicines for the treatment of inflammatory diseases. Adv. Drug Deliv. Rev..

[85] Laroui H., Viennois E., Xiao B., Canup B.S.B., Geem D., Denning T.L., Merlin D. (2014). Fab’-bearing siRNA TNFα-loaded nanoparticles targeted to colonic macrophages offer an effective therapy for experimental colitis. J. Control. Release.

[86] Wang X., Peng J., Cai P., Xia Y., Yi C., Shang A., Akanyibah F.A., Mao F. (2024). The emerging role of the gut microbiota and its application in inflammatory bowel disease. Biomed. Pharmacother..

[87] Liu H., Zhang Y., Zhang M., Yu Z., Zhang M. (2023). Oral administration of platinum nanoparticles with SOD/CAT cascade catalytic activity to alleviate ulcerative colitis. J. Funct. Biomater..

[88] Zhuo Z., Guo K., Luo Y., Yang Q., Wu H., Zeng R., Jiang R., Li J., Wei R., Lian Q. (2024). Targeted modulation of intestinal epithelial regeneration and immune response in ulcerative colitis using dual-targeting bilirubin nanoparticles. Theranostics.

[89] Zhang M., Xu C., Liu D., Han M.K., Wang L., Merlin D. (2018). Oral delivery of nanoparticles loaded with ginger active compound, 6-shogaol, attenuates ulcerative colitis and promotes wound healing in a murine model of ulcerative colitis. J. Crohns Colitis.

[90] Liu H., Cai Z., Wang F., Hong L., Deng L., Zhong J., Wang Z., Cui W. (2021). Colon-targeted adhesive hydrogel microsphere for regulation of gut immunity and flora. Adv. Sci..

[91] Araki T., Mitsuyama K., Yamasaki H., Morita M., Tsuruta K., Mori A., Yoshimura T., Fukunaga S., Kuwaki K., Yoshioka S. (2021). Therapeutic potential of a self-assembling peptide hydrogel to treat colonic injuries associated with inflammatory bowel disease. J. Crohns Colitis.

